# Longer Hospitalizations and Higher In-Hospital Mortality for Acute Heart Failure during the COVID-19 Pandemic in Larger vs. Smaller Cardiology Departments: Subanalysis of the COV-HF-SIRIO 6 Multicenter Study

**DOI:** 10.31083/j.rcm2309292

**Published:** 2022-08-24

**Authors:** Małgorzata Ostrowska, Michał Kasprzak, Wioleta Stolarek, Klaudyna Grzelakowska, Jacek Kryś, Aldona Kubica, Piotr Adamski, Przemysław Podhajski, Eliano Pio Navarese, Edyta Anielska-Michalak, Oliwia Matuszewska-Brycht, Andrzej Curzytek, Aneta Dudek, Leszek Gromadziński, Paweł Grzelakowski, Leszek Kamiński, Andrzej Kleinrok, Marcin Kostkiewicz, Marek Koziński, Paweł Król, Tomasz Kulawik, Gleb Minczew, Marcin Mindykowski, Agnieszka Pawlak, Janusz Prokopczuk, Grzegorz Skonieczny, Bożena Sobkowicz, Sergiusz Sowiński, Sebastian Stankala, Paweł Szymański, Andrzej Wester, Przemysław Wilczewski, Stanisław Bartuś, Andrzej Budaj, Robert Gajda, Mariusz Gąsior, Marcin Gruchała, Jarosław Droźdź, Miłosz Jaguszewski, Piotr Jankowski, Jacek Legutko, Maciej Lesiak, Przemysław Leszek, Przemysław Mitkowski, Jadwiga Nessler, Anna Tomaszuk-Kazberuk, Agnieszka Tycińska, Tomasz Zdrojewski, Jarosław Kaźmierczak, Jacek Kubica

**Affiliations:** ^1^Collegium Medicum, Nicolaus Copernicus University, 85-094 Bydgoszcz, Poland; ^2^Department of Cardiology, Marian Zyndram-Kościałkowski Ministry of Interior and Administration Hospital, 15-471 Białystok, Poland; ^3^Department of Cardiology, Chair of Cardiology and Cardiac Surgery, Medical University of Lodz, 92-213 łódź, Poland; ^4^Department of Cardiology, Hospital of the Ministry of Interior and Administration, 35-111 Rzeszów, Poland; ^5^1st Department of Cardiology, Collegium Medicum, Jan Kochanowski University, 25-736 Kielce, Poland; ^6^Department of Cardiology and Internal Medicine, School of Medicine, Collegium Medicum, University of Warmia and Mazury, 11-041 Olsztyn, Poland; ^7^Department of Cardiology and Cardiac Surgery, 10th Military Hospital and Polyclinic, 85-681 Bydgoszcz, Poland; ^8^Department of Cardiology Independent Public Healthcare in Przeworsk, 37-200 Przeworsk, Poland; ^9^Institute of Humanities and Medicine, Academy of Zamosc, 22-400 Zamość, Poland; ^10^Cardiology Department, Medical Care Center, 37-500 Jaroslaw, Poland; ^11^Department of Cardiology and Internal Diseases, Institute of Maritime and Tropical Medicine, Medical University of Gdansk, 81-519 Gdynia, Poland; ^12^Department of Cardiology, Tertiary Care Hospital, 06-400 Ciechanów, Poland; ^13^Department of Cardiology, Masovian Rehabilitation Center “STOCER”, Dr Włodzimierz Roefler Hospital, 05-800 Pruszków, Poland; ^14^Department of Cardiology, District Hospital, 89-500 Tuchola, Poland; ^15^Department of Cardiology, Dr. Emil Warmiński Tertiary Care Municipal Hospital, 85-808 Bydgoszcz, Poland; ^16^Department of Invasive Cardiology, Central Clinical Hospital of the Ministry of Interior and Administration, 02-507 Warsaw, Poland; ^17^Mossakowski Medical Research Institute, Polish Academy of Sciences, 02-106 Warsaw, Poland; ^18^Department of Cardiology, Polish Hospitals, 47-200 Kędzierzyn-Koźle, Poland; ^19^Department of Cardiology and Intensive Cardiac Care Unit, District Polyclinic Hospital, 87-100 Toruń, Poland; ^20^Department of Cardiology, Medical University of Białystok, 15-276 Białystok, Poland; ^21^Department of Cardiology and Cardiac Intensive Care, Tertiary Care Municipal Hospital, 87-100 Toruń, Poland; ^22^Cardiology Subdivision of Heart Failure. St. Elizabeth Hospital, 48-210 Biała, Poland; ^23^Department of Cardiology, Interventional Cardiology and Electrophysiology with Cardiac Intensive Care Unit, Tertiary Care Hospital, 86-300 Grudziądz, Poland; ^24^1st Department of Physiology, Institute of Medical Sciences, University of Opole 2, Cardiology Center of Kluczbork SCANMED SA, 46-203 Kluczbork, Poland; ^25^Department of Cardiology, Polish Hospitals, 82-400 Sztum, Poland; ^26^2nd Department of Cardiology, Collegium Medicum, Jagiellonian University, 30-688 Cracow, Poland; ^27^Department of Cardiology, Center of Postgraduate Medical Education, Grochowski Hospital, 04-073 Warsaw, Poland; ^28^Department of Kinesiology and Health Prevention, Jan Dlugosz University in Częstochowa, 42-200 Częstochowa, Poland; ^29^Gajda-Med District Hospital in Pultusk, 06-100 Pułtusk, Poland; ^30^3rd Department of Cardiology, Silesian Center for Heart Diseases, Faculty of Medicine in Zabrze, Medical University of Silesia, 41-800 Zabrze, Poland; ^31^1st Department of Cardiology, Medical University of Gdansk, 80-952 Gdańsk, Poland; ^32^Department of Internal Medicine and Geriatric Cardiology, Centre of Postgraduate Medical Education, 00-416 Warsaw, Poland; ^33^Department of Interventional Cardiology, Institute of Cardiology, Jagiellonian University Medical College, John Paul II Hospital in Krakow, 31-202 Cracow, Poland; ^34^Department of Cardiology, Poznan University of Medical Sciences, 61-848 Poznań, Poland; ^35^Department of Heart Failure and Transplantology, National Institute of Cardiology, 04-628 Warsaw, Poland; ^36^Department of Coronary Artery Disease and Heart Failure, Institute of Cardiology, Jagiellonian University Medical College, 31-202 Cracow, Poland; ^37^Department of Arterial Hypertension and Diabetology, Medical University of Gdansk, 80-952 Gdańsk, Poland; ^38^Department of Cardiology, Pomeranian Medical University, 71-899 Szczecin, Poland

**Keywords:** acute heart failure, COVID-19, hospitalization, in-hospital mortality

## Abstract

**Background:**

The coronavirus disease-2019 (COVID-19) pandemic is surging across Poland, leading to many direct deaths and underestimated collateral damage. We aimed to compare the influence of the COVID-19 pandemic on hospital admissions and in-hospital mortality in larger vs. smaller cardiology departments (i.e., with ≥2000 vs. <2000 hospitalizations per year in 2019).

**Methods:**

We performed a subanalysis of the COV-HF-SIRIO 6 multicenter retrospective study including all patients hospitalized in 24 cardiology departments in Poland between January 1, 2019 and December 31, 2020, focusing on patients with acute heart failure (AHF) and COVID-19.

**Results:**

Total number of hospitalizations was reduced by 29.2% in larger cardiology departments and by 27.3% in smaller cardiology departments in 2020 vs. 2019. While hospitalizations for AHF were reduced by 21.8% and 25.1%, respectively. The length of hospital stay due to AHF in 2020 was 9.6 days in larger cardiology departments and 6.6 days in smaller departments (*p *< 0.001). In-hospital mortality for AHF during the COVID-19 pandemic was significantly higher in larger vs. smaller cardiology departments (10.7% vs. 3.2%; *p *< 0.001). In-hospital mortality for concomitant AHF and COVID-19 was extremely high in larger and smaller cardiology departments accounting for 31.3% vs. 31.6%, respectively.

**Conclusions:**

During the COVID-19 pandemic longer hospitalizations and higher in-hospital mortality for AHF were observed in larger vs. smaller cardiology departments. Reduced hospital admissions and extremely high in-hospital mortality for concomitant AHF and COVID-19 were noted regardless of department size.

## 1. Introduction

The coronavirus disease-2019 (COVID-19) pandemic has affected more than 458 million people worldwide. In Poland, the total number of infected patients has reached more than 6 million people accounting for more than 15% of Polish population [1]. There were more than 116 thousand COVID-19 deaths with mortality rate of about 1.9%. The COVID-19 pandemic has become the most challenging public healthcare emergency of our times. Limited availability of specialist care together with omnipresent fear of getting infected with severe acute respiratory syndrome coronavirus 2 (SARS-CoV-2) have contributed to reduction in hospital admissions due to acute cardiovascular conditions [2, 3, 4, 5, 6, 7, 8]. From the Polish perspective, there were reports of decline in-hospital admissions due to various cardiovascular emergencies, together with reduced number of coronary angiographies, percutaneous coronary interventions, both transthoracic and transesophageal echocardiographic examinations, as well as electrotherapy and electrophysiology procedures [9, 10, 11, 12, 13, 14, 15, 16]. Postponing the seek for medical attention due to various cardiovascular emergencies, such as acute coronary syndrome or acute heart failure (AHF), is associated with worse prognosis or can lead to death [17, 18, 19, 20]. Patients with AHF require urgent in-hospital diagnostics and treatment, while those presenting with more advanced symptoms (higher New York Heart Failure [NYHA] class) have worse prognosis [21]. Clinical presentation of AHF and COVID-19 includes acute dyspnea, bringing another diagnostic challenge. Yet, the co-occurrence of AHF and COVID-19 is associated with high mortality [22, 23, 24, 25].

In our recently published Impact of COVID-19 pandemic on acute Heart Failure admissions and mortality: multicentre COV-HF-SIRIO 6 study, we have found that the COVID-19 pandemic has led to reduced hospital admissions for AHF with a significantly lower number of self-referred AHF patients and a higher number of AHF patients brought by an ambulance [26]. Furthermore, the mortality rate for AHF during the COVID-19 era was significantly increased, particularly for concomitant AHF and COVID-19.

We performed this subanalysis of the COV-HF-SIRIO 6 study aiming to compare the influence of the COVID-19 pandemic on hospital admissions and in-hospital mortality in cardiology departments hospitalizing equal or more vs. less than 2000 patients yearly.

## 2. Materials and Methods

The COV-HF-SIRIO 6 study was a retrospective analysis of hospital records of consecutive patients hospitalized in 24 cardiology departments in Poland in 2019 and 2020 [26]. We took under investigation all patients hospitalized from January 1, 2019 to December 31, 2020 (in pre-COVID-19 vs. COVID-19 eras). In this subanalysis of the COV-HF-SIRIO 6 study, we arbitrary divided all cardiology departments into two groups based on the total number of hospitalizations in 2019 (pre-COVID 19 era), with smaller departments defined as those hospitalizing less than 2000 patients, and larger departments—2000 patients or more. We focused on patients hospitalized due to AHF (International Statistical Classification of Diseases and Related Health Problems codes for heart failure I50.x), as well as those with concomitant SARS-CoV-2 infection. The diagnosis of AHF was based on the definition provided by the 2016 European Society of Cardiology guidelines for the diagnosis and treatment of acute and chronic heart failure [27]. The COV-HF-SIRIO 6 study was conducted in accordance with the Declaration of Helsinki and was approved by the Local Ethics Committee (study approval reference number KB 353/2021).

Statistical analysis was carried out using Statistica 13.0 (TIBCO Software Inc., California, USA). Continuous variables were presented as means with standard deviations. Due to non-normal distribution of the investigated data, as demonstrated by the Shapiro-Wilk test, non-parametric tests were used for statistical analysis. Comparisons of continuous variables between two groups were performed with the Mann-Whitney unpaired rank sum test. Comparisons between year 2019 and 2020 were performed with the Wilcoxon signed test. Categorical variables are presented as number and percentage and were compared using the χ^2^ test. Results were considered significant at *p *< 0.05.

## 3. Results

**Fig. 1.**
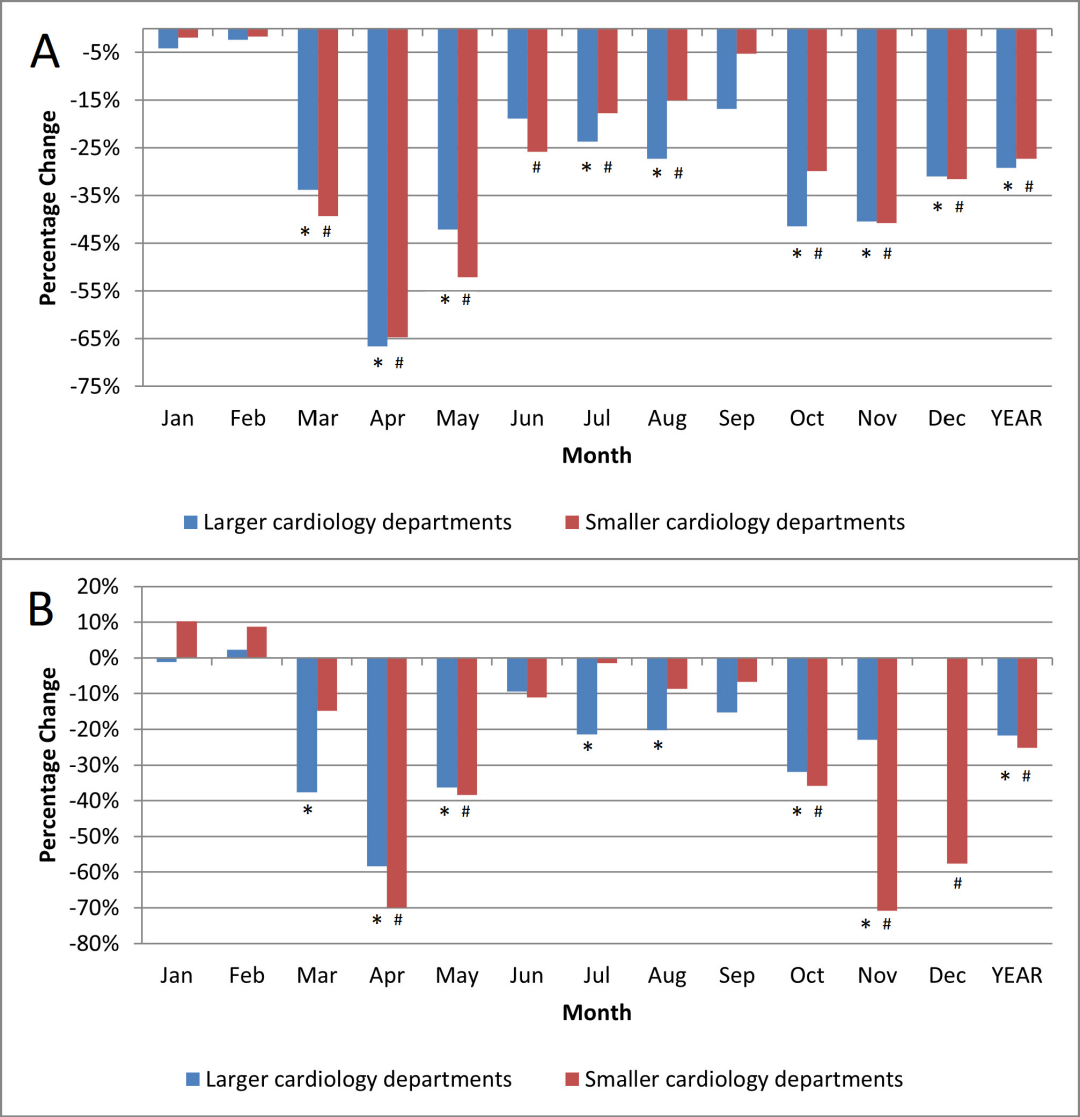
**Hospitalizations percentage change**. (A) Reduction of all-cause hospitalizations during the COVID-19 pandemic in 2020 vs. 2019. (B) Reduction of acute heart failure hospitalizations during the COVID-19 pandemic in 2020 vs. 2019. * *p *< 0.05 for the comparison 2020 vs. 2019 in larger cardiology departments; # *p *< 0.05 for the comparison 2020 vs. 2019 in smaller cardiology departments. Statistical test used: Wilcoxon signed test.

A total of 101,433 patients were hospitalized in 24 cardiology departments in Poland between January 1, 2019 and December 31, 2020. The study cohort was divided into two subgroups including 69,019 patients hospitalized in larger cardiology departments (n = 12) and 32,414 patients hospitalized in smaller cardiology departments (n = 12). The total number of hospitalizations was reduced by 29.2% (*p *< 0.0001) in larger cardiology departments in 2020 as compared with 2019, while smaller departments noted a 27.3% decrease (*p *< 0.0001) in the number of admitted patients in 2020 as compared with 2019 (Fig. 1A).

The number of patients hospitalized for AHF was reduced by 21.8% (*p *< 0.0001) in larger cardiology departments and by 25.1% (*p *< 0.0001) in smaller cardiology departments (Fig. 1B). Interestingly, the reduction of hospitalizations due to AHF was more pronounced following the waves of the pandemic in smaller cardiology centers, while in larger ones it was more consistent throughout the year.

**Fig. 2.**
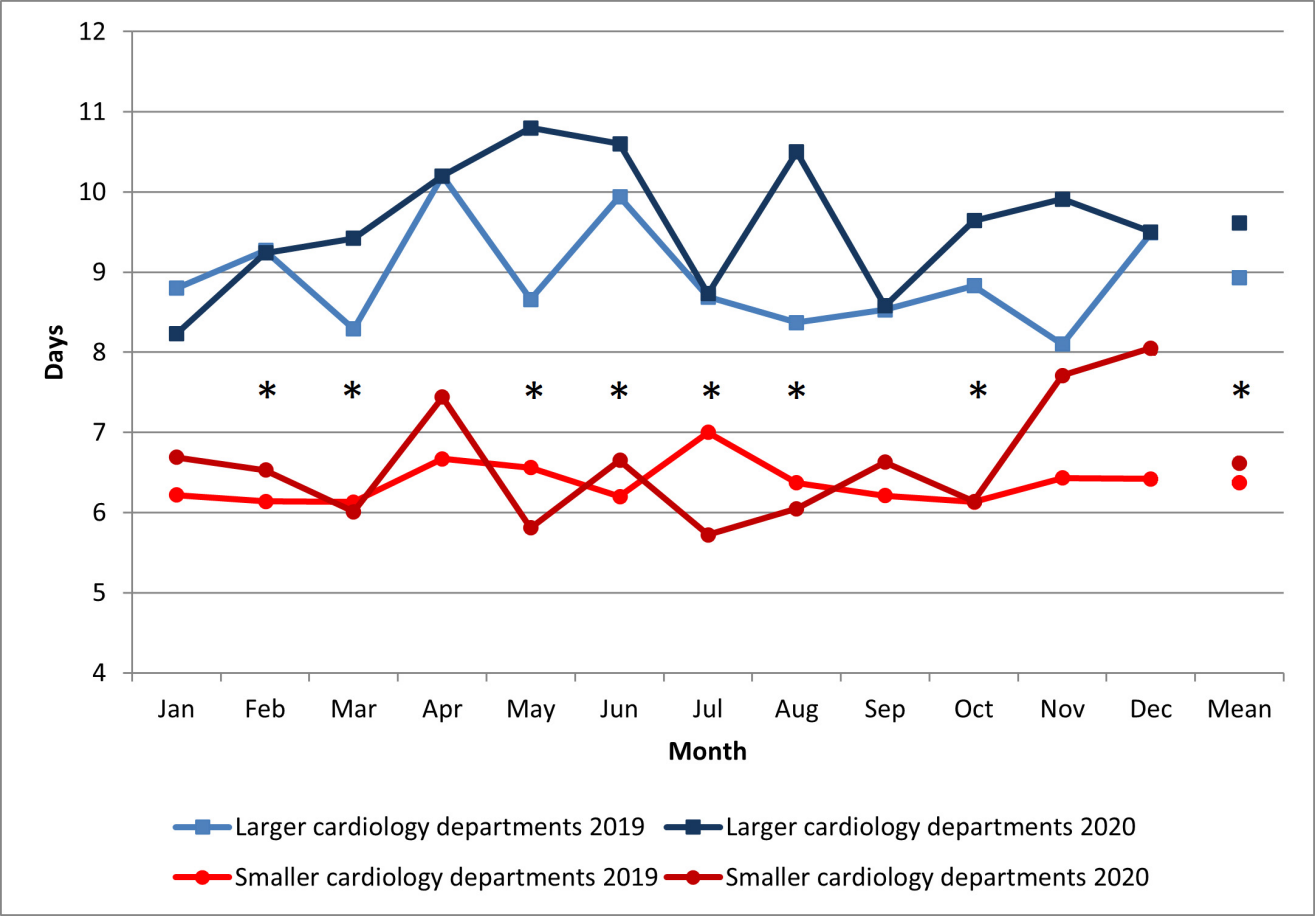
**The length of hospital stay**. * *p *< 0.05 for the comparison larger vs. smaller cardiology departments in 2020. Statistical test used: Mann-Whitney unpaired rank sum test.

Duration of hospital stay due to AHF during the COVID-19 pandemic in 2020 was significantly longer in larger vs. smaller cardiology departments (9.6 vs. 6.6 days; *p *< 0.0001) (Fig. 2).

Regardless of department size, the number of self-referrals was lower in 2020 vs. 2019, with significantly more patients brought by an ambulance (Table 1). However, this trend of decreased number of self-referrals and increased number of patients brought by an ambulance, in smaller cardiology wards visibly followed the peaks of the pandemic, having more random distribution in larger cardiology departments.

**Table 1.** **Modes of hospital admissions**.

Month	Self-reffered										Brought by an ambulance									
	Larger cadiology departments					Smaller cadiology departments					Larger cadiology departments					Smaller cadiology departments				
	2019		2020		*p*	2019		2020		*p*	2019		2020		*p*	2019		2020		*p*
	N	%	N	%		N	%	N	%		N	%	N	%		N	%	N	%	
Jan	131	45.5	150	50.5	0.22	261	62.3	309	66.9	0.15	154	53.5	145	48.8	0.26	111	26.5	109	23.6	0.32
Feb	136	50.4	145	50.7	0.94	239	62.9	280	67.8	0.15	134	49.6	140	49.0	0.87	89	23.4	106	25.7	0.46
Mar	167	49.4	86	42.2	0.10	241	61.6	213	64.0	0.52	171	50.6	117	57.4	0.12	117	29.9	96	28.8	0.75
Apr	148	51.6	47	38.8	0.02	282	63.9	69	51.9	0.01	139	48.4	73	60.3	0.03	110	24.9	51	38.3	0.003
May	150	51.0	92	47.9	0.50	267	67.4	163	66.8	0.87	138	46.9	97	50.5	0.44	92	23.2	65	26.6	0.86
Jun	119	46.9	97	41.1	0.20	241	63.9	206	61.5	0.50	132	52.0	124	52.5	0.90	98	26.0	88	26.3	0.02
Jul	161	55.5	105	44.9	0.02	248	61.7	252	63.6	0.57	126	43.4	125	53.4	0.02	105	26.1	89	22.5	0.23
Aug	136	47.6	95	45.9	0.72	257	64.1	235	64.2	0.97	148	51.7	111	53.6	0.68	105	26.2	100	27.3	0.72
Sep	131	51.6	108	47.8	0.41	247	63.7	244	67.4	0.28	120	47.2	117	51.8	0.32	99	25.5	90	24.9	0.84
Oct	188	60.6	104	49.8	0.01	321	68.6	156	52.0	<0.001	121	39.0	105	50.2	0.01	101	21.6	117	39.0	<0.001
Nov	155	53.4	106	47.7	0.20	263	66.6	46	40.0	<0.001	132	45.5	115	51.8	0.16	102	25.8	44	38.3	0.009
Dec	135	52.1	131	50.0	0.63	243	64.8	77	48.4	<0.001	121	46.7	128	48.9	0.63	108	28.8	63	39.6	0.01
Year	1757	51.4	1266	47.0	<0.001	3110	64.3	2250	62.2	0.04	1636	47.8	1397	51.8	0.002	1237	25.6	1018	28.1	0.009

Statistical test used: χ^2^ test.

The total number of patients with concomitant AHF and COVID-19 was 160 (4.1% of AHF patients hospitalized in 2020) for larger vs. 79 (2.2% of AHF patients hospitalized in 2020) for smaller cardiology departments.

**Fig. 3.**
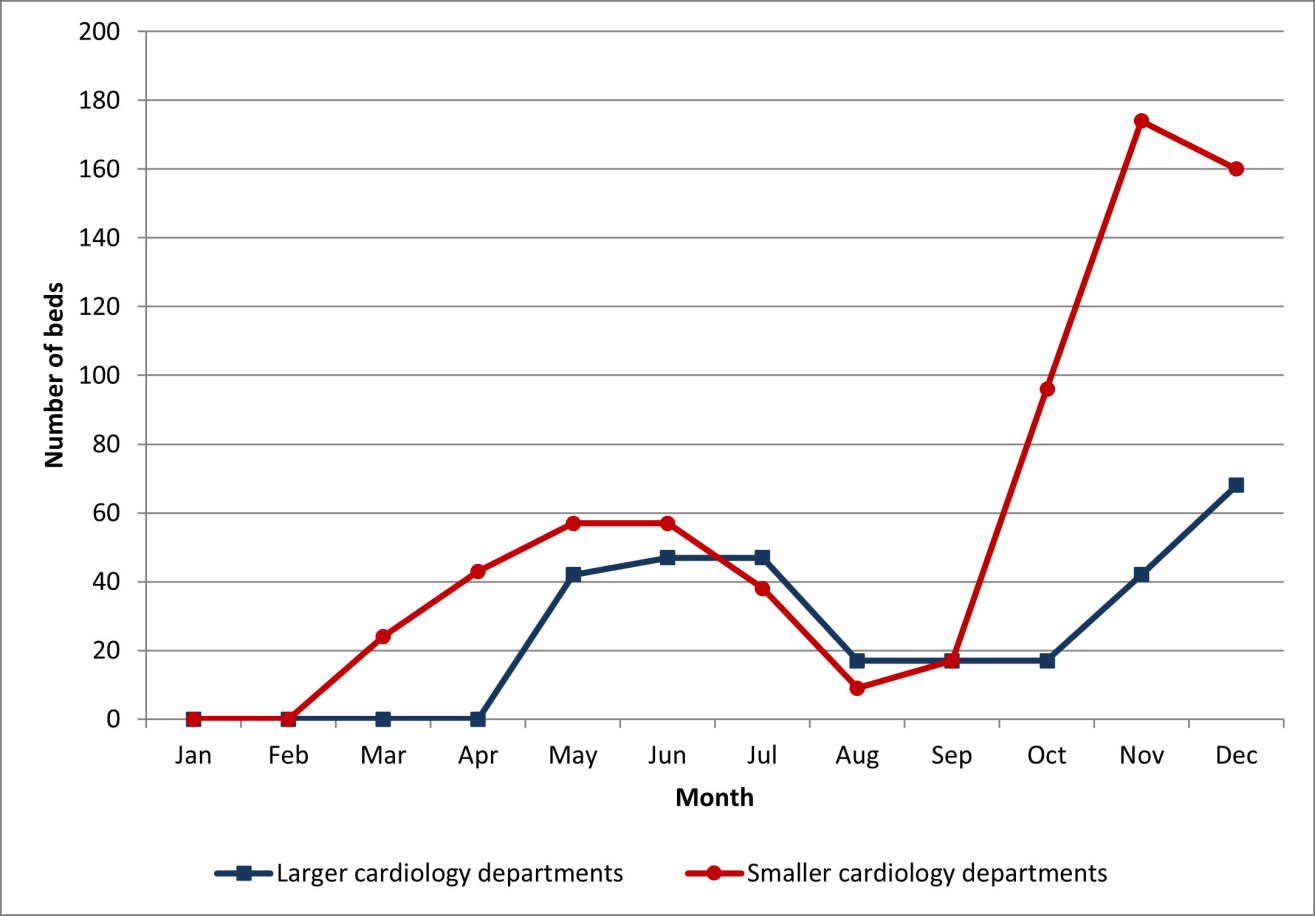
**Number of beds dedicated for COVID-19 patients in 2020**.

Additional beds dedicated for patients with COVID-19 were created in the majority of hospitals, however smaller departments provided more extra beds to treat COVID-19 patients as compared with larger cardiology departments (675 vs. 481 beds) (Fig. 3).

**Fig. 4.**
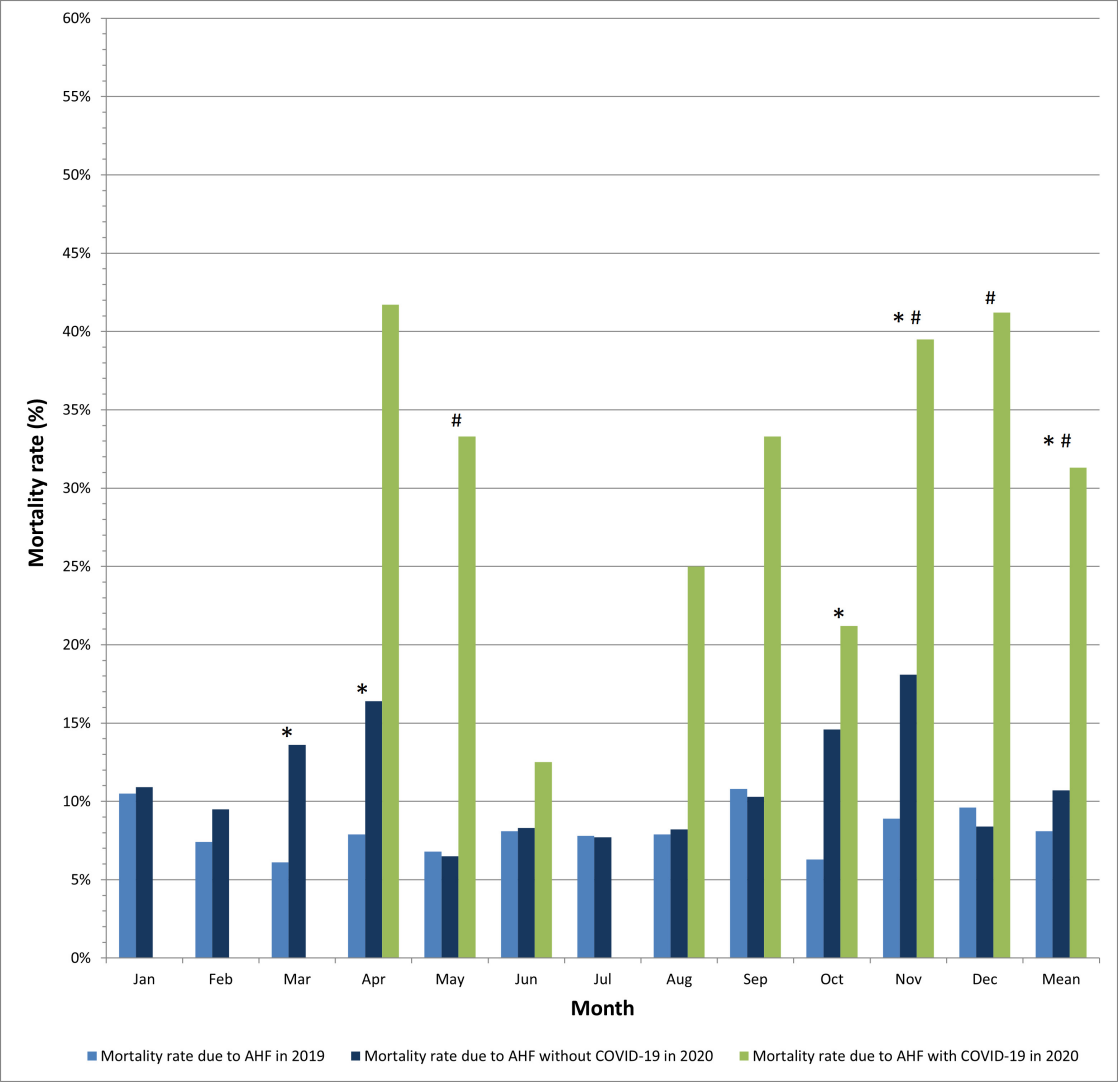
**In-hospital mortality due to acute heart failure in patients with and without COVID-19 in larger cardiology departments**. * *p *< 0.05 for the comparison 2020 vs. 2019; # *p *< 0.05 for the comparison COVID-19 vs. non-COVID-19 in 2020. Statistical test used: χ^2^ test.

In-hospital mortality rate for AHF was significantly higher during the COVID-19 era vs. pre-COVID-19 era, amounting to 10.7% vs. 8.1% (*p* = 0.0004) in larger cardiology departments. The mortality peaks closely followed peaks of the pandemic, with the maximum value of 18.1% in the very peak of the pandemic in November 2020 (Fig. 4).

**Fig. 5.**
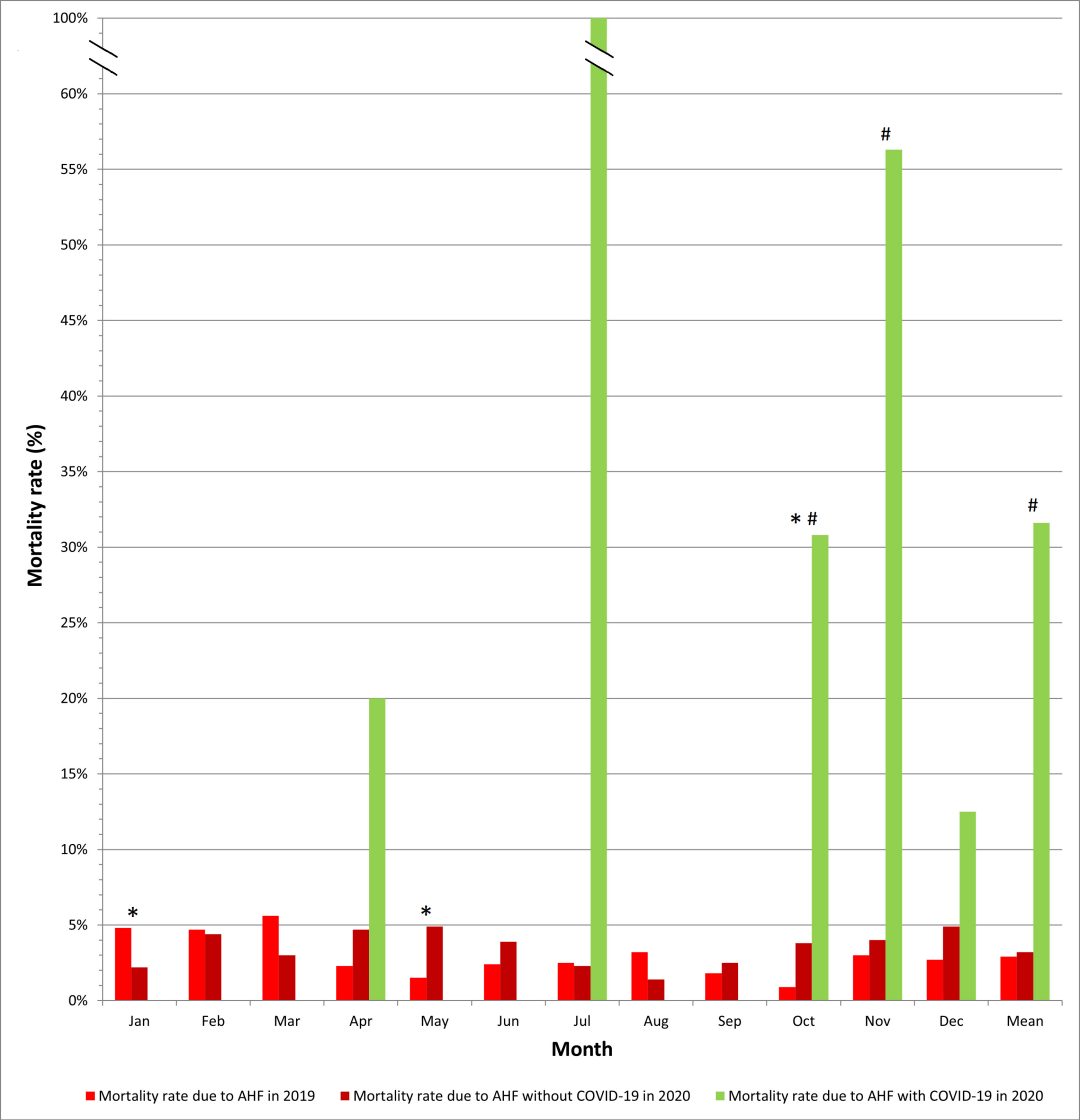
**In-hospital mortality due to acute heart failure in patients with and without COVID-19 in smaller cardiology departments**. * *p *< 0.05 for the comparison 2020 vs. 2019; # *p *< 0.05 for the comparison COVID-19 vs. non-COVID-19 in 2020. Statistical test used: χ^2^ test.

Interestingly, the mortality rate for AHF in smaller cardiology departments did not differ significantly in COVID-19 vs. pre-COVID-19 eras (3.2% vs. 2.9%, *p* = 0.47) (Fig. 5).

In-hospital mortality for AHF during the COVID-19 pandemic was substantially higher in larger vs. smaller cardiology departments (10.7% vs. 3.2%; *p *< 0.0001) (Table 2).

**Table 2.** **In-hospital mortality due to acute heart failure in 2020 in larger vs. smaller cardiology departments**.

Month	Mortality rate in AHF patients without COVID-19					Mortality rate in AHF patients with concomitant COVID-19				
	Larger cardiology departments		Smaller cardiology departments		*p*	Larger cardiology departments		Smaller cardiology departments		*p*
	N	%	N	%		N	%	N	%	
Jan	35	10.9	10	2.2	<0.001	0	0	0	0	N/A
Feb	29	9.5	18	4.4	0.006	0	0	0	0	N/A
Mar	30	13.6	10	3.0	<0.001	0	0	0	0	N/A
Apr	20	16.4	6	4.7	0.002	5	41.7	1	20.0	0.60
May	13	6.5	12	4.9	0.46	3	33.3	0	0	N/A
Jun	21	8.3	13	3.9	0.01	1	12.5	0	0	N/A
Jul	19	7.7	9	2.3	0.001	0	0	1	100	N/A
Aug	18	8.2	5	1.4	<0.001	1	25.0	0	0	N/A
Sep	24	10.3	9	2.5	<0.001	2	33.3	0	0	N/A
Oct	29	14.6	10	3.8	<0.001	7	21.2	12	30.8	0.36
Nov	35	18.1	4	4.0	<0.001	17	39.5	9	56.3	0.25
Dec	20	8.4	7	4.9	0.20	14	41.2	2	12.5	0.09
**Year**	293	10.7	113	3.2	<0.001	50	31.3	25	31.6	0.95

Abbreviations: AHF, acute heart failure; N/A, not applicable. Statistical test used: χ^2^ test.

In-hospital mortality rate for concomitant AHF and COVID-19 was extremely high both in larger and smaller cardiology departments, accounting for 31.3% vs. 31.6%, respectively (Table 2). In-hospital mortality was highest in the very peaks of the pandemic, reaching the highest value of 56.3% in November 2020 in smaller cardiology wards (the reported mortality rate of 100% is a single patient death, thus should be interpreted with caution).

## 4. Discussion

The COVID-19 pandemic is still surging across the globe. Although we do not know whether the pandemic is with us here to stay and if some temporary solutions become permanent, we need to learn how to function in this new reality and provide the best possible care for all patients. One of the first reports on heart failure management in Polish medical centers during the COVID-19 pandemic came from Lelonek *et al*. [28] showing reduced hospitalizations, domination of teleconsultations over in-person visits and e-prescriptions widespread use during the first three months of the COVID-19 pandemic in both academic and non-academic centers. We went one step further and according to our knowledge performed the largest multicentre study including more than 100,000 patients aiming to assess the influence exerted by the COVID-19 pandemic on hospital admissions and in-hospital mortality, and the first study so far confronting larger cardiology departments hospitalizing ≥2000 patients a year vs. smaller cardiology departments hospitalizing <2000 patients a year. Based on our results, the COVID-19 pandemic seems to hit equally smaller and larger, even academic, cardiology departments—we found a reduction in the total number of hospitalizations, as well as hospitalizations due to AHF in 2020 vs. 2019.

Heart failure was found to be an independent risk factor for death in COVID-19 patients [29]. The reported in-hospital mortality for concomitant heart failure and COVID-19 was extremely high, accounting for 49% in the very beginning of the pandemic (March-May 2020) in a large cardiology center in the USA. In our study, the highest mortality rate for co-existing AHF and COVID-19 in larger departments was 41.7% at the beginning of the pandemic in April 2020. In a systematic review and meta-analysis by Yonas *et al*. [30], in a group of 21,640 COVID-19 patients from 18 studies heart failure was associated with poor outcome (odds ratio [OR] 2.86; 95% confidence interval [CI] 2.07–3.95; *p *< 0.001). Patients with co-existing heart failure and COVID-19 had a higher likelihood of mortality (OR 3.46; 95% CI 2.52–4.75; *p *< 0.001). From the Polish perspective, data coming from the public hospitals of Silesian Province showed in-hospital mortality from concomitant heart failure and COVID-19 to be 35.8% as compared with 25.1% in patients without heart failure [31]. In our nationwide study, we also reported concomitant AHF and COVID-19 to be associated with extremely high mortality rates, independently of the size of the department, accounting for 31.3% in larger vs. 31.6% in smaller cardiology departments.

There are loads of data documenting increased mortality rates for concomitant AHF and COVID-19, however very few reports focus on the collateral damage of the COVID-19 pandemic. We found increased mortality rates for AHF in 2020 as compared with 2019, but only in larger cardiology departments. A single center Italian study by Colivicchi *et al*. [19] performed in a large volume hospital reported a 17.2% in-hospital mortality rate for AHF in the beginning of the pandemic in 2020 as compared with 6.3% in the time-matched period of 2019. Data arriving from two referral hospitals in London, UK, also documented significantly higher in-hospital mortality rates for AHF in 2020 vs. 2019 [32]. Studies confronting data from larger/academic hospitals with smaller/district cardiology wards are lacking. In the Danish nationwide cohort study presenting data of all patients hospitalized in Danish hospitals between January 1, 2019 and March 31, 2020 due to heart failure, there was no statistically significant increase in the mortality rates in the COVID-19 era vs. pre-COVID-19 times [33]. In our study, we did not find any differences in in-hospital mortality rates in smaller departments in 2020 vs. 2019. Several arguments may serve as potential explanation of the observed phenomenon of higher in-hospital mortality in larger vs. smaller cardiology departments. Firstly, cardiology departments defined as larger in our study were very often academic centers offering the highest level of reference and very often providing care for severely-ill patients transferred from smaller, non-academic (provincial, regional or district) hospitals. Secondly, patients with AHF with respiratory failure, requiring mechanical ventilation are usually directly admitted to larger cardiology centers. Thirdly, the mean length of hospital stay was longer in larger vs. smaller cardiology department which indirectly indicates that patients hospitalized in larger cardiology departments might have been more severely ill.

Our analysis on modes of hospital admissions showed an increased number of patients brought by emergency teams and a decreased number of self-referrals in 2020 vs. 2019. However, worth underlining is the fact that although in the COVID-19 era patients were more frequently brought by an ambulance than self-referred, the total number of patients brought by emergency teams was lower in 2020 than in 2019. These observations are in line with reports from the very beginning of the pandemic, presenting a decline in the number of interventions of emergency medical teams [34, 35, 36].

### Limitations

There are some limitations of our study, such as its retrospective character. Moreover, data included in the analysis were derived from hospital electronic databases lacking clinical characteristics of patients or results of additional work-up, thus they were unavailable for the assessment. Worth adding is, that only patients with COVID-19 requiring hospitalization were included in the analysis, thus we do not know if asymptomatic COVID-19 would also influence the prognosis in AHF patients.

## 5. Conclusions

In our subanalysis of the COV-HF-SIRIO 6 multicenter study comparing influence of the COVID-19 pandemic on hospital admissions and mortality rate in larger vs. smaller cardiology departments we found: (1) similarly reduced hospital admissions; (2) higher in-hospital mortality for AHF; (3) longer hospital stays for AHF; (4) similar and extremely high in-hospital mortality for concomitant AHF and COVID-19.
